# High-resolution repeat topography of drifting ice floes in the Arctic Ocean from terrestrial laser scanning

**DOI:** 10.1038/s41597-023-02882-w

**Published:** 2024-01-13

**Authors:** David Clemens-Sewall, Chris Polashenski, Ian A. Raphael, Matthew Parno, Don Perovich, Polona Itkin, Matthias Jaggi, Arttu Jutila, Amy R. Macfarlane, Ilkka S. O. Matero, Marc Oggier, Ronald J. W. Visser, David N. Wagner

**Affiliations:** 1 Thayer School of Engineering at Dartmouth College, Hanover, NH USA; 2 NSF National Center for Atmospheric Research, Boulder, CO USA; 3 USACE-CRREL Fort Wainwright, Fairbanks, AK USA; 4 Solea Energy, Thetford, VT USA; 5 UiT The Arctic University of Norway, Tromso, Norway; 6 WSL Snow and Avalanche Research Institute SLF, Davos, Switzerland; 7 Alfred-Wegener-Institut, Helmholtz-Zentrum für Polar- und Meeresforschung, Bremerhaven, 27570 Germany; 8 Finnish Meteorological Institute, Helsinki, Finland; 9 Svalbard Integrated Arctic Earth Observing System Knowledge Centre, Longyearbyen, Norway; 10 International Arctic Research Center, University of Alaska Fairbanks, Fairbanks, AK USA; 11 GroenDoen, Westerwijtwerd, The Netherlands; 12 CRYOS, School of Architecture, Civil and Environmental Engineering, EPFL, Lausanne, Switzerland

**Keywords:** Physical oceanography, Cryospheric science

## Abstract

Snow and ice topography impact and are impacted by fluxes of mass, energy, and momentum in Arctic sea ice. We measured the topography on approximately a 0.5 km^2^ drifting parcel of Arctic sea ice on 42 separate days from 18 October 2019 to 9 May 2020 via Terrestrial Laser Scanning (TLS). These data are aligned into an ice-fixed, lagrangian reference frame such that topographic changes (e.g., snow accumulation) can be observed for time periods of up to six months. Using *in-situ* measurements, we have validated the vertical accuracy of the alignment to ± 0.011 m. This data collection and processing workflow is the culmination of several prior measurement campaigns and may be generally applied for repeat TLS measurements on drifting sea ice. We present a description of the data, a software package written to process and align these data, and the philosophy of the data processing. These data can be used to investigate snow accumulation and redistribution, ice dynamics, surface roughness, and they can provide valuable context for co-located measurements.

## Background & Summary

Repeat Terrestrial Laser Scanning (TLS) topography measurements were part of the Multidisciplinary drifting Observatory for the Study of Arctic Climate (MOSAiC) expedition, in which researchers aboard *R/V Polarstern*^1^ drifted with and studied the same collection of ice floes in the Central Arctic from October 2019 to May 2020^2–4^. Arctic sea ice has grown dramatically younger and thinner in recent decades^5^ and the overall objectives of MOSAiC were to understand the causes and consequences of this ‘new Arctic’. To do so, researchers were divided into teams studying the snow and ice (including on-ice and satellite remote sensing)^2^, atmosphere^3^, ocean^4^, ecosystem, and biogeochemistry. We conducted the TLS measurements as part of the ice team, for the primary purpose of quantifying snow accumulation and redistribution. Other applications of these data include observations of ice dynamics; surface roughness for ice-atmosphere interactions; and providing context for atmospheric observations, remote sensing instruments (e.g., on-ice radars^6^), autonomous buoys, snow pit measurements, and more.

Snow substantially affects the Arctic sea ice mass balance due to its opposing impacts of insulating in the winter (restraining ice growth) and reflecting shortwave radiation in the summer (protecting against ice melt^7^). Three of the four most important uncertainties impacting September sea ice volume in the CICE sea ice model^8^ are related to the thermal and optical properties of the snow^9^. Snow spatial variability due to wind driven snow redistribution impacts these properties^10,11^. However, redistribution is challenging to measure due to this spatial variability. Repeat observations of snow changes on a substantial area of the same piece of ice are needed to observe these redistribution processes and understand their mechanisms. Furthermore, the magnitude of snow accumulation can be very small. For example, the largest snowfall event on MOSAiC precipitated just 1.6 cm water equivalent^12^. Thus, our observations of changes must be highly accurate. Finally, to observe snow accumulation and redistribution throughout the winter, measurements must be feasible in polar night.

TLS is routinely used to make highly-accurate measurements of snow accumulation on areas of 40 m^2^ to 0.5 km^2^ ^13–17^. The instrument is a laser scanner that is mounted on a ∼ 2.2 m tall tripod. Due to the generally flat topography of sea ice and shadowing, the instrument collects topographic information up to 100–200 m from itself. To observe a larger area, we relocate the tripod and collect measurements from different locations, which we then co-register into a common reference frame. TLS measurements on Arctic sea ice face unusual challenges and our procedures for the MOSAiC Expedition were informed by prior experiments, including: the Seasonal Ice Zone Observing Network project^18^; the Snow, Wind, and Time project^17^; and the Sea Ice Dynamics Experiment. First, the typical temperatures of −15 to −35 °C are below the operating range of commercially-available TLS instruments. We addressed this issue with a custom-designed heater case. Second, TLS measurements are typically aligned in a geodetic reference frame via highly-accurate GNSS measurements. However, on drifting sea ice, the relevant reference frame for snow processes is a lagrangian reference frame fixed to the surface of the ice. We developed a custom software package—pydar^19^—to align repeat TLS measurements into this lagrangian, ice-fixed reference frame. To verify that our alignment achieved the necessary vertical accuracy for snow accumulation, we statistically validated it through comparison with *in-situ* measurements^20^. The quantitative results from our alignment validation are specific to this dataset. Finally, the TLS data contain potentially-useful information that are irrelevant to our needs (e.g., sub-cm snow surface roughness in areas near the scanner, backscatter reflectance, roughness relevant to aerodynamic drag, etc…). We hope that future researchers will use these data for purposes that we have not imagined. To facilitate this future usage, we have designed pydar to preserve the full scope of the data and to make our data processing decisions transparent to future researchers.

The primary purpose of this manuscript is to describe the TLS data collected at MOSAiC. However, given the unique challenges of using TLS on drifting sea ice, some discussion of lessons learned and future methodological developments is warranted. Prior experience on sea ice near Utqiaġvik, AK^17,18^ found that spacing scan positions between 150 and 200 m apart generally produced acceptable data. However, the ice at MOSAiC was rougher than those experiments, and the placement of scan positions was also restricted due to not trespassing in sensitive measurement sites. Under these constraints, we found that spacing scan positions around 100 m apart produced better data, although we sometimes prioritized having measurements co-located with complementary measurements sites (e.g., the snow and ice thickness transects) over achieving the best TLS coverage. Additionally, the alignment procedures and validation were developed after the expedition, thus the alignment validation relies on what coincident *in-situ* measurements were available and had not experienced blowing snow events between the *in-situ* measurement and the TLS acquisition. The statistical validation methods presented herein are applicable for future campaigns, but the specific quantitative results depend on the measurements. Therefore, validation should be conducted for any TLS campaign on sea ice. For future campaigns, we strongly recommend including more *in-situ* point measurements of snow surface changes coincident with the TLS measurements. One expeditious approach for this would be to include a small array of snow thickness stakes near each reflector post. This would ensure that the *in-situ* measurement sites were visible from multiple scan positions, and the snow surface measurements could be made quickly while distributing the reflectors at the start of a TLS measurement day.

This manuscript describes the repeat TLS data collected on the MOSAiC expedition from October 2019 to May 2020^21^. We present the philosophy of the data processing, and it’s implementation in pydar^19^. Finally, we validate the vertical alignment and discuss considerations for reuse of these data.

## Methods

### Terminology

We use the following terms throughout this manuscript. They are mostly drawn from their usage in RiSCAN (Riegl’s software for acquiring and processing TLS data):

- Scan Position: set-up the tripod at a given location and measure the topography within the scanner’s line of sight.
- SingleScan: the data collected from a single scan position. We use this term to refer to both the point cloud of topographic measurements from this scan position and ancillary data such as the locations of TLS reflectors within the scanner’s reference frame at this scan position and the rigid transformations that register and align this SingleScan with others (see below).
  - Project: a collection of SingleScans covering a contiguous area that were collected during a sufficiently short time interval such that no topographic change occurred between scan positions (sometimes ice deformation occurred during a Project, these exceptions are described in the Usage Notes). Typically, the set of SingleScans in a Project were collected in a single day of measurements although on some occasions measurements were collected over two days.
  - Registration: the act of computing the rigid transformations that describe the spatial relationships between the different SingleScans in a Project. Registration places all SingleScans in a Project into a common reference frame (whose origin and unit vectors are typically defined by an arbitrary SingleScan).
  - Scan Area: a region of ice whose topography we measured over time with a succession of Projects.
  - Alignment: the act of computing the rigid transformations such that SingleScans from different Projects in the same Scan Area are in a common reference frame. Alignment is necessary to precisely locate topographic changes (e.g., how much snow accumulation occurred at specific location on the ice from 4 January to 11 March).

### Data collection

**Fig. 1:**
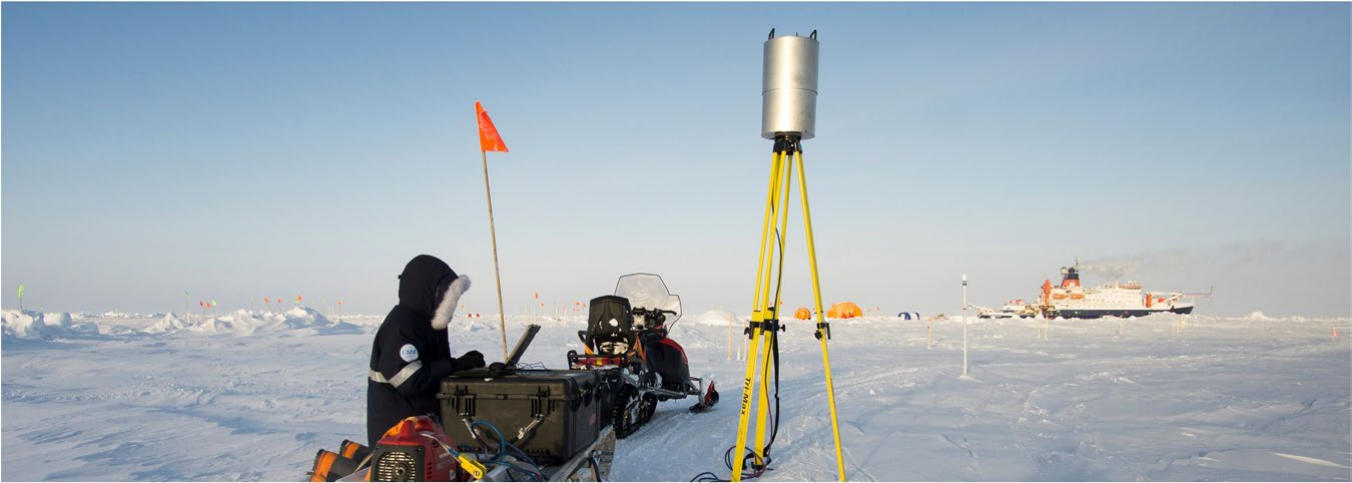
Photo showing data acquisition of a typical scan position. The Riegl VZ1000 terrestrial laser scanner is inside the custom heater case (metal cylinder) on top of the tripod. The researcher controls the scanner from the laptop via WiFi. To the right of the tripod is a Riegl 10 cm cylinder reflector mounted on a post frozen into the ice. The Polarstern can be seen in the background along with other installations of the ice camp including Balloon Town^3^ (orange tents) and Ocean City^4^ (blue and white tent). Photo credit: S. Svavarsdóttir.

**Fig. 2:**
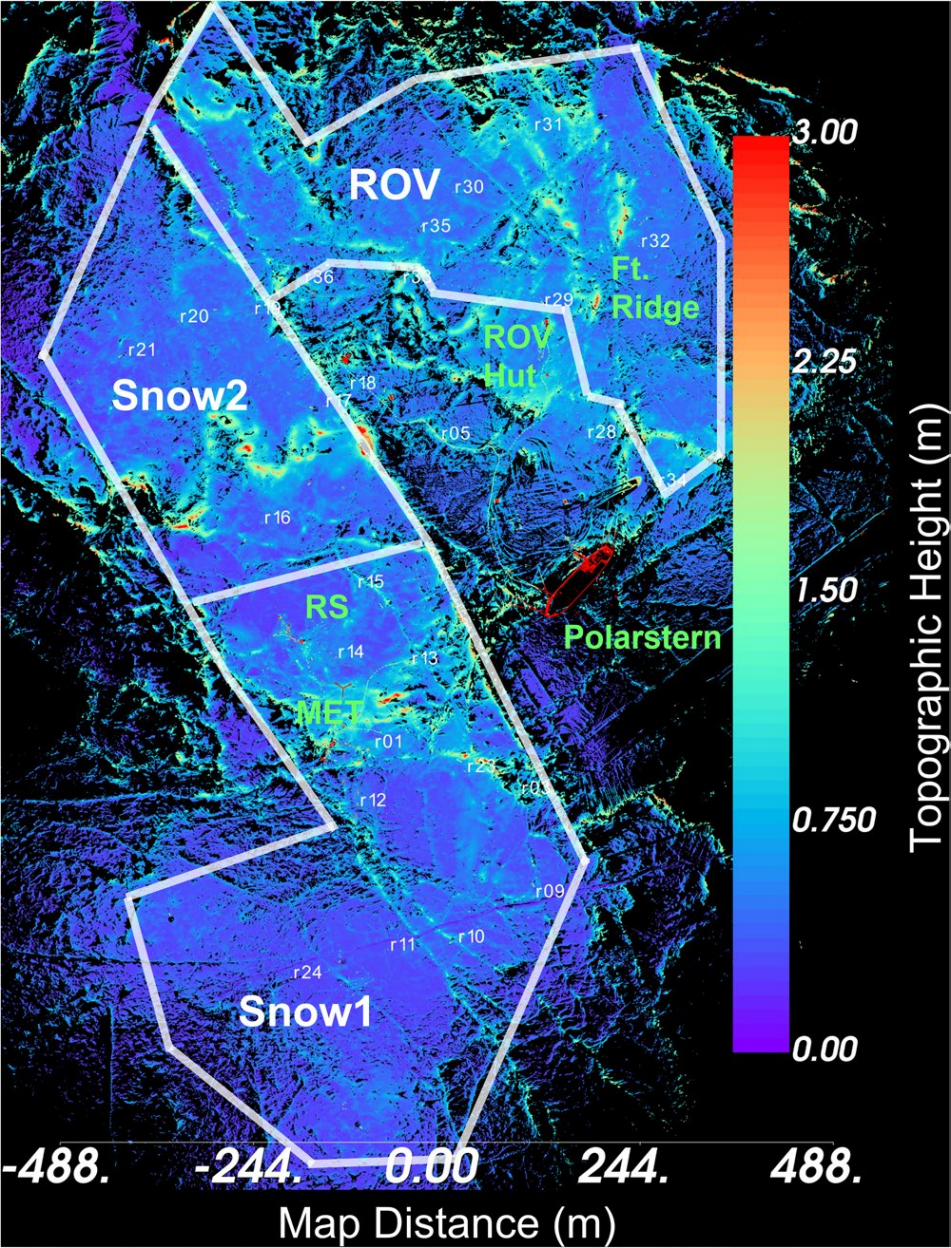
Map of the three Scan Areas in the MOSAiC Central Observatory^2^ in early February. The Scan Areas are labelled and roughly outlined in white (the exact extent of the measured areas changed due to ice deformation and the number of SingleScans conducted). Notable installations are indicated in green (see MOSAiC snow and ice overview publication^2^ for more installations). Reflector locations are also shown in white. Reflector heights are provided in Table 2. This map is a rendering of the combined point cloud data from Projects mosaic_rov_040220.RiSCAN (collected on 4 February) and mosaic_01_060220.RiSCAN (collected on 6–7 February). The origin for topographic height is the surface of recently frozen lead (so the values approximately correspond to height above the sea surface).

We used a Riegl VZ1000, which has an eye-safe, near infrared laser (1550 nm). For each scan position, we mounted the scanner on a tripod, and the scanner rotated on vertical and horizontal axes to create a point cloud of its surroundings. The scanner was controlled via WiFi from a field laptop. The angular stepwidths in the azimuthal and vertical directions were each 0.025° and it took approximately 8 minutes to acquire a point cloud with a Laser Pulse Repitition Rate of 300 kHz. The origin and unit vectors of the point cloud are defined relative to the scanner and this reference frame is named the Scanner’s Own Coordinate System (SOCS). Air temperature was typically below the VZ1000’s minimum operating temperature, so we placed the scanner in a custom-designed heater case (Fig. 1) to maintain its temperature within acceptable bounds. Due to the overall flat topography, occlusions, and low reflectivity of snow and ice at 1550 nm, the scanner collects useful data up to 100–200 m from its location. To map a larger area, on each measurement day we relocated the scanner to additional scan positions and acquired subsequent measurements (i.e., we collected a SingleScan at each scan position), which we then linked together into a Project. Choosing the locations for scan positions was a trade-off between maximizing the area covered, co-locating with other measurements, minimizing shadows within the measured area, and not trespassing in sensitive measurement sites. In order to make a complete map, we registered the SingleScans measured from each scan position into a common reference frame. We placed Riegl 10 cm cylinder reflectors on posts frozen into the ice (Figs. 1, 2), and used them to locate and orient each SingleScan in the Project’s common reference frame (described below in Data Processing in RiSCAN). Typically between 4 and 10 reflectors were visible from each scan position. These reflectors also served as the starting point for aligning Projects collected in the same Scan Area on different days into a lagrangian, ice-fixed reference frame.

### Scan areas

Repeat TLS observations on MOSAiC were focused on three primary Scan Areas: Snow1, Snow2, and ROV (Fig. 2 and Table 1). These Scan Areas were selected to observe a variety of ice topography, to co-locate with other measurements, and to be logistically accessible. In March, ice dynamics caused the Met City atmospheric measurement site^3^ and the on-ice Remote Sensing measurement site^2^ to be removed from the Snow1 scan area (where they had been located from October through February). Additional TLS measurements were made at these sites (which we labelled ‘RS’ for Remote Sensing and ‘MET’ for Met City; Table 1). Below are descriptions of each Scan Area.

**Table 1:** Summary of repeat TLS measurements from October 2019 to May 2020 on MOSAiC.

Project Name	Date	Scan Area(s)	# SingleScans	Location
mosaic_01_101819.RiSCAN	2019-10-18	Snow1	2	84.79°N, 133.09°E
mosaic_01_102019.RiSCAN	2019-10-20	Snow1	5	84.97°N, 132.76°E
mosaic_01_102519.RiSCAN	2019-10-25	Snow1	5	85.44°N, 128.15°E
mosaic_01_110119.RiSCAN	2019-11-01	Snow1	7	85.80°N, 122.97 ° E
mosaic_02_110619.RiSCAN	2019-11-06	Snow2	7	85.93°N, 117.74°E
mosaic_01_110819.RiSCAN	2019-11-08	Snow1	3	85.92°N, 116.53°E
mosaic_02_111319.RiSCAN	2019-11-13	Snow2	8	86.10°N, 117.84°E
mosaic_01_111519.RiSCAN	2019-11-15	Snow1	8	86.16°N, 118.20°E
mosaic_01b_061219.RiSCAN.RiSCAN.RiSCAN	2019-12-06^a^	Snow1, Snow2	12	86.14°N, 122.25°E
mosaic_01_122719.RiSCAN	2019-12-27	Snow1	5	86.68°N, 115.95°E
mosaic_rov_040120.RiSCAN	2020-01-04	ROV	6	86.99°N, 115.44°E
mosaic_01_040120.RiSCAN	2020-01-04^a^	Snow1	7	87.01°N, 115.35°E
mosaic_rov_110120.RiSCAN	2020-01-11	ROV	5	87.21°N, 111.40°E
mosaic_01_180120.RiSCAN	2020-01-18	Snow1	8	87.41°N, 98.27°E
mosaic_rov_190120.RiSCAN	2020-01-19	ROV	8	87.40°N, 98.36°E
mosaic_rov_250120.RiSCAN	2020-01-25	ROV	11	87.41°N, 92.79°E
mosaic_01_290120.RiSCAN	2020-01-29	Snow1	8	87.47°N, 95.18°E
mosaic_rov_040220.RiSCAN	2020-02-04	ROV	9	87.48°N, 95.30°E
mosaic_01_060220.RiSCAN	2020-02-06^a^	Snow1, Snow2	16	87.60°N, 94.04°E
mosaic_01_150220.RiSCAN.RiSCAN	2020-02-15	Snow1	8	88.07°N, 79.87°E
mosaic_rov_220220.RiSCAN.RiSCAN	2020-02-22	ROV	9	88.58°N, 64.54°E
mosaic_01_280220.RiSCAN	2020-02-28	Snow1	7	88.34°N, 33.99°E
mosaic_rov_110320.RiSCAN	2020-03-11	ROV	1	88.27°N, 31.95°E
mosaic_rov_140320.RiSCAN.RiSCAN	2020-03-14	ROV	4	87.67°N, 24.59°E
mosaic_rov_180320.RiSCAN	2020-03-18	ROV	4	87.14°N, 17.28°E
mosaic_01_220320.RiSCAN	2020-03-22	Snow1	6	86.23°N, 15.70°E
mosaic_02_260320.RiSCAN	2020-03-26	Snow2	5	86.00°N, 13.09°E
mosaic_02_300320.RiSCAN.RiSCAN	2020-03-30	Snow2	5	85.38°N, 13.20°E
mosaic_02_040420.RiSCAN	2020-04-04^a^	Snow2, ROV	11	84.67°N, 12.89°E
mosaic_01_080420.RiSCAN	2020-04-08	Snow1	4	84.48°N, 14.64°E
mosaic_01_080420b.RiSCAN	2020-04-08	Snow1	3	84.48°N, 14.64°E
mosaic_02_110420_rov.RiSCAN	2020-04-11	ROV	6	84.33°N, 14.75°E
mosaic_02_130420_2.RiSCAN	2020-04-13	Snow2	6	84.29°N, 14.96°E
mosaic_rov_170420.RiSCAN	2020-04-17	ROV	6	84.41°N, 13.67°E
mosaic_rs_170420.RiSCAN	2020-04-17	RS	1	84.41°N, 13.67°E
mosaic_rov_220420.RiSCAN	2020-04-22	ROV	6	84.13°N, 15.89°E
mosaic_rs_220420.RiSCAN	2020-04-22	RS	1	84.13°N, 15.89°E
mosaic_02_230420.RiSCAN	2020-04-23	Snow2	6	84.08°N, 16.05°E
mosaic_01_250420.RiSCAN.RiSCAN	2020-04-25	Snow1	4	84.00°N, 15.59°E
mosaic_01_260420.RiSCAN	2020-04-26	Snow1	3	83.93°N, 15.51°E
mosaic_rov_290420.RiSCAN	2020-04-29	ROV	7	84.02°N, 17.24°E
mosaic_02_300420.RiSCAN	2020-04-30	Snow2	6	83.94°N, 17.42°E
mosaic_rs_300420.RiSCAN	2020-04-30	RS	1	83.94°N, 17.42°E
mosaic_01_030520.RiSCAN	2020-05-03	Snow1	4	83.89°N, 17.96°E
mosaic_met_040520.RiSCAN	2020-05-04	MET	2	83.91°N, 18.35°E
mosaic_rov_02_090520.RiSCAN	2020-05-09	Snow2, ROV	12	83.79°N, 14.09°E

^a^Some SingleScans were collected on the following day.

**Table 2:** Reflector heights both relative to the local snow surface (Terrain Relative Height) and relative to the approximately the sea surface (Pole Height).

Reflector	Pole Height (m)	Terrain Relative Height (m)
r01	2.10	1.38
r03	2.03	0.99
r05	2.01	1.10
r09	1.72	1.05
r10	1.71	1.13
r11	1.81	1.51
r12	1.79	1.26
r13	2.89	1.67
r14	2.06	1.60
r15	2.47	1.73
r16	1.96	1.57
r17	1.96	1.54
r18	1.93	1.16
r19	2.06	1.56
r20	1.90	1.44
r21	1.73	1.38
r23	1.99	1.46
r24	1.85	1.55
r28	1.80	1.24
r29	1.77	1.30
r30	1.63	1.21
r31	2.36	1.48
r32	1.98	1.53
r33	2.86	1.49
r34	2.84	1.44
r35	2.09	1.53
r36	2.30	1.47

Pole Height is in the same reference frame as Fig. 2.

#### Snow1 Scan Area

The Snow1 Scan Area (hereafter we use ‘Snow1’ to refer specifically to the TLS measurement area) was generally off the bow and port side of Polarstern and composed of residual ice of which only the upper 30 cm was solid when we arrived in October^22^, refrozen melt ponds, first year ice in refrozen leads, and first year ridges. We first measured Snow1 on 18 October and our final measurement was on 3 May. Ice dynamics caused frequent (multiple times per month) crack and ridge formation in the Scan Area. These dynamics resulted in substantial variation in the area of ice measured and the number of SingleScans collected in each Project. Snow1 was co-located with the SLoop snow and ice thickness transect^23^, the Snow1 snow sampling area^2^, the BGC3 ice coring area, the Met City atmospheric measurement site^3^ (18 October to 28 February), the first on-ice Remote Sensing site^2^ (18 October to 15 November), the second on-ice Remote Sensing site^2^ (6 December to 28 February), the Ocean City oceanic measurement site^4^ (18 October to 6 December), the Bow Stakes mass balance site^20^ (6 December to 28 February), the Met Stakes mass balance site^20^ (18 January to 3 May), the Stakes3 mass balance site^20^ (6 December to 26 April), the second Remotely Operated Vehicle site^2^ (1 November to 6 December), and a number of vibrating wire ice stress gauges^2^. The given dates are the first and last dates that the installations were present in the TLS data, not necessarily the installation or decommission dates of the installations.

#### Snow2 Scan Area

The Snow2 Scan Area (hereafter we use ‘Snow2’ to refer specifically to the TLS measurement area) was generally off the bow and starboard side of Polarstern beyond the logistics area^2^ and was composed of refrozen melt ponds and an approximately 1 m tall (on average) second year ridge. No ice thickness measurements were made in Snow2, but the residual ice was likely thicker than that in Snow1. Similar level ice in the NLoop transect had a modal thickness of approximately 75 cm in early November^23^. We first measured Snow2 on 6 November and our final measurement was on 9 May. Snow2 was the most stable region observed. The core of the measurement area was not deformed from 6 November 9 May with the exception of a 1-m-wide crack that formed between 13 November and 6 December. The measurement area progressively shrank due to ice dynamics removing areas in November, March, April, and May. On two occasions—6 December and 6 February—we made measurements on Snow1 and Snow2 as part of the same Project. Snow2 was co-located with the Snow2 snow sampling area^2^, an atmospheric flux chamber measurement site, a section of the NLoop snow and ice thickness transect^23^ (6 November to 6 December), the Stakes1 mass balance site^20^ (only on 6 December, thereafter this was part of the ROV Scan Area), and the Alli’s ridge measurement site^2^ (only on 6 December, thereafter this was part of the ROV Scan Area).

#### ROV Scan Area

The ROV Scan Area (hereafter referred to as ‘ROV’, it was named for the Remotely Operated Vehicle installation) was generally off the stern and starboard side of Polarstern beyond the logistics area^2^ and was composed of deformed second year ice, refrozen melt ponds, level first year ice, and first year ridges. We first measured the ROV Scan Area on 4 January and our final measurement was on 9 May. In early January, the modal ice thickness on both level first year ice^20^ and level second year ice^23^ was approximately 1 m. In March, ice dynamics displaced the first year ice region out of the ROV Scan Area and demolished the Ft. Ridge measurement site. These dynamics also produced young ice in a 40-m-wide, refrozen lead. The core region of ROV was connected to Snow2 from 6 December to 9 May. On two occasions—4 April and 9 May—we made measurements on Snow2 and ROV as part of the same project. ROV was co-located with the third Remotely Operated Vehicle site^2^, the NLoop snow and ice thickness transect^23^, the Ft. Ridge measurement site^2^ (4 January to 18 March), half of the Alli’s Ridge measurement site^2^ (4 January to 22 February), the Ridge Ranch mass balance site^20^ (19 January to 11 March), the Stakes4 mass balance site^20^ (4 January to 22 February), the David’s Ridge measurement site^2^ (14 March to 9 May), the ROV3 broadband albedo transect^24^ (11 April to 9 May), the SYI broadband albedo transect^24^ (29 April to 9 May), and a number of vibrating wire ice stress gauges^2^.

### Data Processing in RiSCAN

TLS data acquisition and initial post-processing steps were conducted in RiSCAN PRO (Riegl’s software for TLS data acquisition and processing: http://www.riegl.com/products/software-packages/riscan-pro/) following standard protocols described in the user manual. The following steps were conducted for each Project. First, an arbitrary SingleScan was chosen to be the origin of the Project. This SingleScan was levelled to establish the horizontal axis plane using the VZ1000’s onboard inclination sensors. Next, another SingleScan was registered to the first by computing the rigid transformation that minimizes the sum of the least-squares error in the positions of pairs of reflectors (a.k.a. ‘keypoints’) observed in both SingleScans. We repeated this process until all SingleScans in the Project are registered. Finally, we refined the registration of each SingleScan except for the origin with RiSCAN’s ‘Multi-Station Adjustment’. In this process, meter-scale planar facets were extracted from each SingleScan. Then, pairs of overlapping facets and pairs of reflectors between the SingleScans were all used as keypoints and an optimization procedure adjusted the rigid transformations of each SingleScan (except the origin) in order to minimize the sum of the least-squares error between all keypoints. Unlike in urban environments where walls, roads, and other human-made objects provide large planar facets, planar surfaces at MOSAiC were mostly meter-scale or smaller wind-scoured snow surfaces. When conducting Multi-Station Adjustment, we found that using a search radius (maximum distance between potential keypoints) between 0.3 and 0.7 m produced the best results. Search radii larger than this tended to match planar facets that were not truly co-planar (e.g., different faces of a snow dune), which causes misalignment.

The data for each Project was stored in a directory with the same name as the Project. All relevant parameters were exported from RiSCAN into open formats. The rigid transformation for each SingleScan is represented by a 4 × 4 matrix (using homogeneous coordinates^25^) that is named the Scanner’s Own Position (SOP) matrix. The SOP matrices for each SingleScan were exported into tab-delimited. DAT files in the Project directory (e.g., ‘ScanPos001.DAT’). We gave each reflector a unique identifier (e.g., ‘r01’) that was consistent across all of the Projects. We exported the reflector positions (in the Project’s reference frame) for each Project into a comma-delimited file (named ‘tiepoints.csv’) in the Project directory. Finally, we exported the point cloud data itself for each SingleScan in LAS 1.4 format into a subdirectory named ‘lasfiles’. LAS 1.4 (https://github.com/ASPRSorg/LAS) is an open, community standard for point cloud data that is maintained by the American Society for Photogrammetry and Remote Sensing.

### Data processing in pydar

#### Overview of pydar

The overall objective of pydar is to align SingleScans from different Projects in the same Scan Area into a lagrangian, ice-fixed reference frame such that one can observe topographic changes (e.g., snow deposition and erosion) over time. Furthermore, we sought to facilitate re-use of these data by preserving features of the data even when they are unimportant for our use case (e.g., cm-scale point density near the scanner, backscatter reflectance data, etc…) and enabling future researchers to revise our alignment of SingleScans (e.g., if they design a superior alignment procedure). This section provides a brief description of key features of pydar^19^ and the steps taken to process the repeat TLS data from MOSAiC. Additional functionality and implementation details can be found in the code documentation. To achieve these goals, pydar has an object-oriented design that mimics the hierarchical structure of the TLS data. And, pydar distinguishes between the spatial relationships of TLS points measured from the same scan position (i.e., within a SingleScan) and the spatial relationships of TLS points measured from different scan positions (i.e., different SingleScans in a Project). pydar is implemented primarily in Python^26^, with substantial use of the Numpy^27^, Scipy^28^, and VTK^29^ libraries. Some functionality is implemented in Cython^30^.

The core of pydar consists of four related classes: SingleScan, Project, ScanArea, and TiePointList (in this manuscript we use teletype font to reference classes and methods in code). SingleScan objects store the point cloud data for that SingleScan in the Scanner’s Own Coordinate System (as a vtkPolyData object: SingleScan.polydata_raw) and the rigid transformation to transform that point cloud into the desired reference frame (as a vtkTransform object: SingleScan.transform). Separating the spatial information for the point cloud of an entire Project into SingleScans enables us to adjust the spatial relationships between points from different SingleScans (as we do below) without altering the spatial relationships within each SingleScan. SingleScan also contains methods for filtering the point cloud data (e.g., FlakeOut^31^) and reading and writing the data. When we filter TLS points, we set flags in the ‘Classification’ data field (following LAS 1.4 conventions: https://github.com/ASPRSorg/LAS), rather than deleting points. A Project object contains the set of SingleScan objects for this Project (as a dictionary: Project.scan_dict) and an object representing the reflector positions (an instance of TiePointList). Project also contains methods for visualizing the data (e.g., Project.display_project), writing data output (e.g., Project.write_las_pdal), and converting the point cloud into a surface representation (e.g., Project.point_to_grid_average_image). Finally, a ScanArea object contains a set of Project objects for this Scan Area (as a dictionary: ScanArea.project_dict) and methods for aligning the SingleScans within those Projects (e.g., ScanArea.z_tilt_alignment_ss, see below).

#### Filtering of TLS data

Wind-blown snow particles were filtered using FlakeOut^31^ with the standard parameters (z_max = 3, radial_precision = 0.005, z_std_mult = 3.5, and leafsize = 100) and assigned the classification flag ‘65’ (the LAS 1.4 standard prescribes that user-defined classifications be greater than 63). Additionally, we manually filtered the logistics area (classification flag ‘73’) from the Snow2 and ROV data because vehicle traffic there substantially disturbed the snow surface. For TLS data that has been processed by pydar, we store the processed data in a subdirectory of the Project directory named ‘npyfiles_archive’. Within the ‘npyfiles_archive’ subdirectories there are subdirectories for each SingleScan (e.g., ‘ScanPos001’). These subdirectories contain numpy^27^ files for the point locations in the Scanner’s Own Coordinate System (‘Points.npy’) and each data attribute (e.g., ‘Reflectance.npy’, ‘Classification.npy’, etc…). We decided to store the data in this manner for three reasons. First, ‘.npy’ is a space-efficient, open-source format (https://numpy.org/doc/stable/reference/generated/numpy.lib.format.html) which is easy to open with widely-available tools. Second, separating the data attributes allows the user to only load the attributes they need into memory, which is useful because the ‘.las’ file for a SingleScan is approximately 600 MB. Third, reading ‘.npy’ files into memory in Python^26^ is considerably faster than reading ‘.las’, which speeds up the overall workflow.

#### Alignment of TLS data

For our purposes of observing snow accumulation and redistribution, the greatest source of error is bias due to misalignment of SingleScans from measurements on different dates. The stochastic errors due to measurement uncertainty within a SingleScan are insignificant^15^. We developed a three step process to align SingleScans from one Project (Project_1) to another Project (Project_0):

1. Coarsely align Project_1 to Project_0 by minimizing the least-squares error between their reflector positions.
2. Align each SingleScan in Project_1 to the nearest SingleScan in Project_0 using local maxima as keypoints, which reduces tilt biases (a tilt bias of 0.0001 radian creates a vertical error of 0.01 m at 100 m distance from the scanner).
3. Perform a fine-scale vertical alignment for each SingleScan in Project_1 by minimizing modal vertical differences between it and Project_0.

For reflector alignment, we labeled each reflector with a consistent name (e.g., ‘r01’) in RiSCAN. Ice deformation and errors in the VZ1000’s reflector search process can shift a reflector relative to the other reflectors. We manually compared the pairwise distances between reflectors and used only the set of reflectors whose pairwise distances between Projects changed by 0.02 m or less. This aligned the scans horizontally to within 0.02 m. Typically, this set comprised 4 to 8 reflectors. With this set of reflectors as keypoints, we computed the rigid transformation that minimized the least-squares error between the keypoints^32^ (implemented in TiePointList.calc_transformation). The default version of this rigid transformation calculation (mode = 'LS' in TiePointList.calc_transformation) requires at least 3 pairs of keypoints and has six degrees of freedom: translations in the 3 spatial directions and rotations around the 3 unit vectors (roll, pitch, and yaw). However, sometimes when there were just 3 or 4 pairs of keypoints, small vertical errors in the reflector positions produced unrealistic tilts, assessed manually by looking at the vertical differences between the aligned Projects. For these cases and when there were only 2 pairs of keypoints, we calculated the rigid transformation without permitting tilt changes (mode = 'Yaw' in TiePointList.calc_transformation). Finally, in two, cases ice dynamics caused there to be no reflectors whose pairwise distance changed by less than 0.02 m. In these cases, we used a single reflector to determine the translational components of the rigid transformation and manually adjusted the yaw component such that flag posts frozen into the ice (see Fig. 1 for example) aligned to within 0.02 m at their bases. Manual inspection of the results indicated that reflector alignment brings vertical biases within 0.05 m and tilt biases within 0.001 radian.

Local maxima alignment for each SingleScan followed the same process as reflector alignment, except that it used local maxima as keypoints instead of reflectors. Local maxima are mostly the crests of ridges, hummocks, or human installations (e.g., poles) and are unlikely to erode or accumulate snow. A pair of local maxima from the SingleScans was required to be within a set spatial tolerance of each other to be used as keypoints. We defined the tolerance in cylindrical coordinates centered on the scanner’s location of the SingleScan that was being aligned. We used a tolerance of 0.0008 radian yaw, 0.001 radian tilt, and 0.1 m radial difference. Local maxima were located on 5 × 5 m regions (changing the region size to 2 × 2 m or 10 × 10 m had no discernable impact on the results). These settings produced several hundred keypoints for each pair of SingleScans from the different Projects. The tilt biases after local maxima alignment were less than 0.0001 radian (i.e., a 1 cm vertical offset across a 100 m distance), determined by manual inspection. Local maxima alignment is implemented in ScanArea.max_alignment_ss.

Finally, for the fine-scale vertical alignment, we exploited the fact that numerous field observations at MOSAiC suggested that a plurality of the snow surface did not change on weekly, or even monthly, time-frames. These observations included: snowmobile tracks did not typically get covered by snow except for isolated, distinct snow drifts. The indentations made by the feet of the TLS tripod were often visible when we revisited scan position locations. The circular mark left in the snow by the atmospheric flux chamber measurement could be identified months after the measurement was made. Cm-scale micro-relief on the snow surface observed near the TLS scanner (where point density is very high) appears consistent between scans, unless a snow dune happened to form in that location. Certain distinctive snow features (e.g., barchan dunes) remained unchanged for months. If the plurality of the snow surface does not change between Projects, then the modal vertical difference must be zero. For each SingleScan, we computed the distribution of vertical differences between it and the Project we were aligning it to. We used a raster with 1 m horizontal resolution created by averaging the z-components of the TLS points within each grid cell^16^. We used only grid cells with at least 25 points per square meter in the SingleScan and the Project it was being aligned to. The vertical component of the transformation for the SingleScan was set such that the modal difference is zero. Fine-scale vertical alignment is implemented in ScanArea.z_alignment. Manual inspection of the results indicated that vertical biases were reduced to within about 0.01 m (see below for Technical Validation).

In Snow1, deformation occurred frequently and throughout the Scan Area, such that there was no core ice floe with multiple reflectors on it that did not experience ice deformation for an extended period of time (as there was for Snow2 and ROV). Alignment steps 2 and 3 are predicated on the ice floe itself remaining the same, and hence could not be applied in this dynamic environment. We include Snow1 for completeness and for use by future researchers (e.g., these ice dynamics would not prevent another researcher from using the Snow1 data to compute aerodynamic drag coefficients). However, we did not validate its vertical alignment to cm-scale accuracy nor do we recommend that the data be used for snow accumulation without further work quantifying ice deformation (which is beyond the scope of this data processing).

For convenience and to avoid needing to recompute the transformations, the rigid transformation aligning each SingleScan has been written out to a ‘.npy’ file that can be loaded directly in pydar. In the Project’s directory, there is a subdirectory named ‘transforms’, within which there are subdirectories for each SingleScan (e.g., ‘ScanPos001’). The transformation is in this subdirectory and is named ‘current_transform.npy’. Finally, because the ROV and Snow2 Scan Areas were connected, we decided to place them within the same lagrangian, ice-fixed reference frame (for which the origin happens to be near the Remotely Operated Vehicle tent^2^).

### Surface Reconstruction from Point Clouds

Many applications of topographical data—including measurement of snow accumulation—require surfaces or gridded data rather than point clouds (the format of TLS data). To produce gridded surfaces, we used gaussian process regression^33^. Also known as kriging, gaussian process regression is an interpolation technique that provides the best linear unbiased estimate (minimizes least-squares error) of a parameter (e.g., surface height) at unsampled locations (e.g., a regular grid of points) given nearby measurements (e.g., TLS points) and the covariance function (also referred to as the kernel)^33,34^. It has previously been applied to TLS data on Arctic sea ice^35^. The vertical uncertainty in an individual TLS data point increases with distance from the scanner due to the divergence of the laser beam (0.3 mrad for the VZ1000) and is represented as gaussian noise^15^. Because the TLS collects useful data up to 200 m from the scanner, the vertical uncertainties in individual data points varies by an order of magnitude. One advantage of gaussian process regression, is that it can factor in the vertical uncertainty of each point when interpolating.

We chose to use an exponential covariance function because it is the simplest covariance function that can represent continuous, non-differentiable (i.e., not-smooth) surfaces^34^. We chose this because wind-driven spatially variable snow deposition and erosion produce rough snow bedforms on horizontal scales of 10 cm to several meters^36^. The exponential covariance function contains two hyperparameters: the ‘range’, defined as the distance at which the correlation between two points is less than 5%; and the ‘sill’, defined as the variance between two uncorrelated points (i.e., points further apart than the range). We can estimate appropriate values of the hyperparameters from the data itself, by optimizing the marginal likelihood of the gaussian process^34^. On the scale of our scan areas (several hundred meters across) the snow and ice topography varies from rough areas of pressure ridges and rubble to smooth areas on level ice. To account for this spatial variability, we divided the domain into a grid of non-overlapping 1.2 m by 1.2 m subdomains. For each subdomain we estimated the sill from the variance of TLS points within a 5 m radius of the center of the subdomain and the range via marginal likelihood optimization using GPyTorch^37^ with a KeOps^38^ kernel on an NVIDIA Quaddro P2000 GPU. The size of the subdomains was chosen such that they were significantly smaller than the spatial scales of the pressure ridges (which were at least 10 s of meters) and balancing GPU memory limitations with computational time. After estimating the hyperparameters, we interpolated the gaussian processes on a regular grid with 10 cm spacing. This surface reconstruction process is implemented in Project.merged_points_to_image.

## Data Records

Repeat TLS data in Table 1 are available at the Arctic Data Center^21^. The top level of the archive contains a directory for each Scan Area: Snow1, ROV (which includes the Snow2 projects since they are in the same reference frame), RS (April Remote Sensing site), and MET (single Project focused on Met City on 4 May). Within these directories is a subdirectory for each Project that contains all data records for that Project (as described in the Methods section). An illustrative directory tree for a Scan Area is shown below.

## Technical Validation

### Alignment Validation

We qualitatively assessed the alignment results by examining the patterns of snow accumulation and redistribution in comparison with the locations of the scan positions. In general, after the full alignment process we did not find artifacts due to either the distance from the nearest scan position or on regions observed from different scan positions. In contrast such artifacts were readily-apparent when conducting only reflector alignment, indicating that local maxima and fine-scale vertical alignment steps improved the alignment for observing snow processes. To quantitatively assess the uncertainty in our alignment procedure, we used a Bayesian statistical model to compare changes in snow thickness measured by TLS with manual measurements of snow thickness changes at snow thickness stakes in the Ridge Ranch mass balance site^39^, while accounting for the uncertainties in the individual measurements. The Ridge Ranch mass balance site was located on level, first-year ice within the ROV Scan Area from 19 January to 18 March (at which point ice deformation relocated Ridge Ranch outside of the TLS measurement area). Ridge Ranch included nine snow thickness stakes, arranged in a cross, with approximately eight meters between stakes. Each stake was frozen into the ice and had a metric length scale marked on its side. Snow thickness was measured by manually observing the location of the snow surface on this metric scale with an accuracy of 0.01 m. Changes in snow thickness at each stake can be determined by comparing repeat measurements. Because the stake was permanently frozen into the ice, these measurements directly recorded changes in the snow surface height. They are unaffected by the changes in snow or snow-ice interface properties that may change the penetration depth of a snow thickness probe^40^. Manual measurements at Ridge Ranch were made on 28 January, 5 February, and 7 March. ROV TLS measurements were made on 25 January, 4 February, and 11 March (Table 1). Observers in the field did not observe snow accumulation or drifting snow at Ridge Ranch during 25–28 January, 4–5 February, and 7–11 March. From these three TLS measurements and three manual stakes measurements, we defined two evaluation periods (Table 3) in which the TLS and the manual measurements should observe the same change in the surface at each stake, if there were no measurement noise or bias due scan misalignment.

**Table 3:** Evaluation periods for validating TLS alignment.

Evaluation Period	First TLS Measurement	Second TLS Measurement	First Manual Measurement	Second Manual Measurement
1	2020-01-25	2020-02-04	2020-01-28	2020-02-05
2	2020-02-04	2020-03-11	2020-02-05	2020-03-07

We define *S*_*k,i*_ as the change in the snow surface observed by manual stake measurements at stake *i* over a evaluation period *k*. Mathematically, *S*_*k,i*_ is the true change in the snow surface *r*_*k,i*_ plus noise due to measurement error *n*_*k,i*_ (Eq. 1). We consider the measurement accuracy of an individual stake reading (0.01 m) to represent two standard deviations of the measurement noise. This implies that the standard deviation of the measurement noise for a single stake reading is 0.005 m. Thus, we represent *n*_*k,i*_ as an instance of a zero-mean, normally distributed random variable with a variance ${\sigma }_{s}^{2}=2* {(0.005m)}^{2}$. Note that each *S*_*k,i*_ is the difference of two independent measurements, hence the multiplication by two in the variance. We define *t*_*k,i*_ as the change in the snow surface observed by TLS for each stake and evaluation period. *t*_*k,i*_ is the true change *r*_*k,i*_ plus measurement noise *m*_*k,i*_ minus a constant bias for the evaluation period *b*_*k*_ due to scan misalignment (Eq. 3). To quantify the change observed in the TLS data at a stake, we looked at the mean vertical distance for all exclusive pairs of horizontally closest points within 10 cm of the stake (excluding the stake itself) in the scans at the beginning and end of the evaluation period. The measurement noise *m*_*k,i*_ is represented as an instance of zero-mean, normally distributed random variable with a variance (${\sigma }_{t,k,i}^{2}$) determined by the vertical uncertainty in each TLS point due to the laser beam spreading with distance^15^.

1 $${s}_{k,i}={r}_{k,i}+{n}_{k,i}$$

2 $${n}_{k,i} \sim N\left(0,{\sigma }_{s}^{2}\right)$$

3 $${t}_{k,i}={r}_{k,i}+{m}_{k,i}-{b}_{k}$$

4 $${m}_{k,i} \sim N\left(0,{\sigma }_{t,k,i}^{2}\right)$$

To quantify how misaligned our TLS measurements may be, we computed the posterior distribution of the bias, *b*_*k*_, given our measurements at each stake. We define *y*_*k,i*_ as the difference between the stake measurement *s*_*k,i*_ and the TLS measurement *t*_*k,i*_ for each stake and evaluation period (Eq. 5). *y*_*k,i*_ is equal to the bias for the evaluation period *b*_*k*_ plus the difference in the measurement noise for each measurement, which we denote by *g*_*k,i*_. The measurement noise for the TLS and stake measurements are independent and normally distributed, allowing us to represent their difference as a zero-mean, normally distributed random variable whose variance is the sum of the variance of each measurement noise. Thus, each difference *y*_*k,i*_ is an instance of a normally distributed random variable given by:

5 $${y}_{k,i}={s}_{k,i}-{t}_{k,i}={b}_{k}+{g}_{k,i} \sim N({b}_{k},{\sigma }_{g,k,i}^{2})$$

6 $${g}_{k,i}={n}_{k,i}-{m}_{k,i} \sim N(0,{\sigma }_{g,k,i}^{2})$$

7 $${\sigma }_{g,k,i}^{2}={\sigma }_{s}^{2}+{\sigma }_{t,k,i}^{2}$$

Applying Bayes Rule, the posterior probability density of *b*_*k*_ given the data for an evaluation period $p({b}_{k}|{y}_{k})$ is proportional to the prior probability density $p({b}_{k})$ multiplied by the likelihood $p({b}_{k}|{y}_{k})$ (Eq. 8). *y*_*k*_ denotes the set of all observations for evaluation period *k*: ${y}_{k}=\{{y}_{k,1},{y}_{k,2},...,{y}_{k,N}\}$. Equation 5 indicates that the likelihood of a single observation is gaussian. Conditioned on the bias, the observations are independent. Hence the likelihood of all observations is their product (Eq. 9). Finally, we chose to represent our prior for the bias as a normal distribution with a mean of zero (we do not expect there to be any bias) and a variance of ${\tau }_{0}^{2}={(0.02m)}^{2}$. This variance was chosen because a bias of greater than 0.04 m (twice the standard deviation of our prior) would be obvious on manual inspection of the data, and was not observed. Moreover, the variance of the uncertainty in the observations is lower than the variance of this prior. So increasing the variance of the prior has little impact on the results (i.e., the information content of the observations is considerably higher than this prior). This yields the following expression for the posterior probability density:

8 $$p({b}_{k}|{y}_{k})\propto p({b}_{k})p({y}_{k}|{b}_{k})$$

9 $$\propto \quad p({b}_{k})\mathop{\prod }\limits_{i=1}^{N}p({b}_{k}|{y}_{k})$$

10 $$\propto \quad exp\left(-\frac{{b}_{k}^{2}}{2{\tau }_{0}^{2}}\right)\mathop{\prod }\limits_{i=1}^{N}exp\left(-\frac{1}{{\sigma }_{g,k,i}^{2}}{({y}_{k,i}-{b}_{k})}^{2}\right)$$

Algebraic simplification of Eq. 10 yields that the posterior density $p({b}_{k}|{y}_{k})$ is a gaussian (Eq. 11) whose mean is the mean of the prior and the observations, each weighted by the inverse of their variance (Eq. 12). The variance of $p({b}_{k}|{y}_{k})$ is determined from the sum of the inverse variances of the prior and the observations (Eq. 13).

11 $$p({b}_{k}|{y}_{k})=N({\mu }_{k},{\tau }_{k}^{2})$$

12 $${\mu }_{k}={\tau }_{k}^{2}\mathop{\sum }\limits_{i=1}^{N}\frac{{y}_{k,i}}{{\sigma }_{g,k,i}^{2}}$$

13 $$\frac{1}{{\tau }_{k}^{2}}=\frac{1}{{\tau }_{0}^{2}}+\mathop{\sum }\limits_{i=0}^{N}\frac{1}{{\sigma }_{g,k,i}^{2}}$$

We use the posterior density of the bias to establish a 95% credible interval (the interval between the 2.5th and 97.5th percentiles in the distribution of a parameter) for the bias in our alignment procedure.

**Fig. 3:**
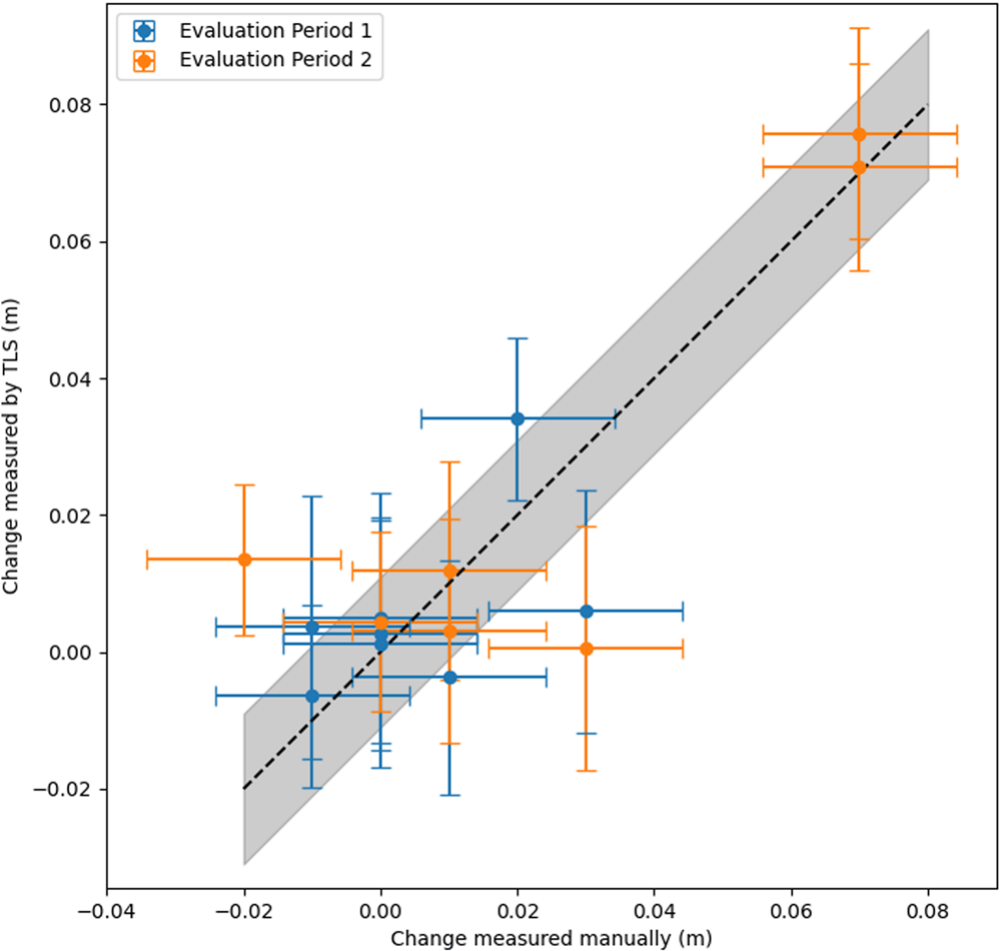
Comparison of snow surface changes measured manually at snow thickness stakes with the snow surface changes at the same stakes measured by TLS during two evaluation periods (Table 3). Error bars show the 95% confidence intervals of the measurement uncertainty for the manual measurement (Eq. 2) and the TLS measurement (Eq. 4, note that each TLS measurement has an individual uncertainty which depends on the distance of the stake from the scanner and the number of TLS points within 10 cm of the stake). The black, dashed line is the 1:1 line (on which the manual measurement is equal to the TLS measurement). The grey shading above and below line represents the ± 0.011 m uncertainty in our posterior estimate of the bias. No bias between the manual and TLS measurements is visually apparent.

To validate the TLS alignment, we compared the snow surface change observed by TLS and manual measurements (Fig. 3) at the Ridge Ranch mass balance site^41^ during evaluation periods 1 and 2 (Table 3). Each evaluation period included at least one snow accumulation and redistribution event. The evaluation periods collectively span 25 January to 11 March. Manual measurements indicated that most stakes experienced little to no change in the snow surface in either evaluation period. The largest snow accumulation observed manually at any of the stakes occurred during evaluation period 2, when 0.07 m of snow accumulated at two stakes (Fig. 3). The TLS measurements, too, observed approximately 0.07 m of change at the same two stakes and little to no change at the others (Fig. 3). With these data, we compute the posterior density of the bias (Table 4) following Eqs. 1–12. The estimated mean biases for evaluation period 1 and 2 are −0.001 m and −0.004 m, respectively. And the minimum and maximum 95% credible interval bounds are −0.011 m and 0.007 m respectively. Thus, we conclude that the bias due to scan misalignment is less than 0.011 m. We stress that these numerical values are particular to the ROV and Snow2 scan areas at MOSAiC (regular ice deformation in the Snow1 area will require future work to correct for). Future TLS measurement campaigns should conduct *in-situ* validation measurements for their specific measurement sites.

**Table 4:** Scan misalignment bias.

Evaluation Period	Expectation of Posterior Density of Bias	95% Credible Interval
1	−0.001 m	[−0.009 m, 0.007 m]
2	−0.004 m	[−0.011 m, 0.004 m]

### Surface Reconstruction Validation

**Fig. 4:**
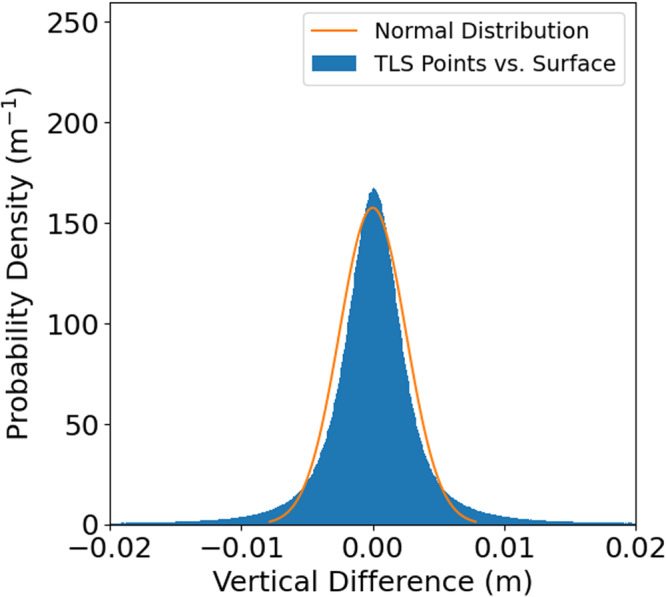
Histogram of vertical differences between TLS data points and reconstructed surface for a 250 m x 65 m region of sea ice containing a large ridge and level ice measured on 26 March 2020. The overlain curve shows a normal distribution with the same median absolute deviation as the data. The 2.5th percentile to the 97.5th percentile of the differences roughly matches a normal distribution but the tails are more extreme.

We validated our gaussian process regression approach to surface reconstruction by examining the differences between the vertical components of TLS data points and the reconstructed surface. Figure 4 shows an example of the distribution of these differences for a 250 m × 65 m region reconstructed on a 10 cm grid containing a large ridge and level ice. The mean difference is 2.5 × 10^−5^ m, the median difference is 6.5 × 10^−5^ m, the standard deviation of the differences is 0.0054 m and the median absolute deviation, a metric of the variability of data that is robust to extreme values^42^, for these data is 0.0017 m. This is similar in magnitude to the median standard deviation of the vertical uncertainty due to laser beam divergence: 0.0026 m. These results combined with manual inspection of the differences suggest that almost all of the differences between the TLS points and the reconstructed surface can be attributed to the divergence of the laser beam with distance from the scanner. A small fraction (less than 5%) of the differences are due to areas with high surface roughness on horizontal length scales of less than the 10 cm grid spacing. These rough areas cause the tails of the distribution of differences to be more extreme than a normal distribution (Fig. 4). For the purpose of assessing snow accumulation, the uncertainties in our surface reconstruction approach are insignificant compared to the alignment uncertainties (0.011 m, see above). However, we caution that applications involving surface roughness may want to further develop surface reconstruction techniques.

## Usage Notes

On 18 March, the ice was deforming within the ROV Scan Area while we were collecting TLS data. On 8 April, Snow1 split in two while we were collecting TLS data. Data collected after the deformation began are in a second project (‘mosaic_01_080420.RiSCAN’). We recommend caution when using data collected during deformation events. The convention in project names was inadvertently switched from ‘MMDDYY’ to ‘DDMMYY’ at the turn of the year (except for ‘mosaic_01b_061219.RiSCAN.RiSCAN.RiSCAN’ on 6 December). A function is provided in pydar to convert a Project’s name to its date (pydar.mosaic_date_parser). Sometimes extra ‘.RiSCAN’s were included in the Project name, these have no significance.

Researchers interested in extending the functionality of pydar (e.g., adding feature tracking functionality for ice deformation, point cloud segmentation, etc) are encouraged to contact the corresponding author in case related efforts are underway. We also welcome discussions on potential uses of these data and collaborations with other data products.

## Data Availability

pydar is available at Zenodo^19^ (https://zenodo.org/record/8120858).
